# Mental Health Stressors and Barriers to Support Among Rural Farmers: A Qualitative Study of Agricultural Communities in the East South-Central United States

**DOI:** 10.3390/ijerph23080983

**Published:** 2026-07-29

**Authors:** Jeanne M. Ward, Melissa Perkins, John R. Blosnich

**Affiliations:** 1 School of Nursing, Indiana University, 600 Barnhill Drive, Indianapolis, IN 46202, USA; 2 Suzanne Dworak-Peck School of Social Work, University of Southern California, 669 W 34th St., Los Angeles, CA 90089, USAblosnich@usc.edu (J.R.B.); 3 Center for Health Equity Research and Promotion, VA Pittsburgh Healthcare System, Pittsburgh, PA 15240, USA

**Keywords:** suicide, burden, agriculture, prevention, remote

## Abstract

**Highlights:**

**Public health relevance—How does this work relate to a public health issue?**

- Farmers face elevated suicide risk and chronic occupational stressors, including financial stressors, long hours, and climate and weather uncertainty.
- The study examines how farmers may not manifest their distress, alongside strong coping narratives.

**Public health significance—Why is this work of significance to public health?**

- The resourcefulness of farmers helps them function, but it can also mask their distress and delay seeking help.
- Cultural and structural factors in agriculture need to be considered, including stigma, privacy concerns, self-reliance, and trust.

**Public health implications—What are the key implications or messages for practitioners, policy makers and/or researchers in public health?**

- Rural health practitioners should frame conversations around realities of agriculture and social determinants rather than querying mental health symptoms.
- Future interventions should be farmer-centered and privacy-minded, addressing structural issues such as financial, environmental, and access-related stressors.

**Abstract:**

**Introduction:** Suicide rates among individuals working in agriculture remain disproportionately high in the United States. Although farming-related stressors and coping strategies are documented, gaps remain in understanding how farmers sustain themselves psychologically amid chronic stress and why elevated suicide risk persists despite apparent resilience. **Purpose:** This study sought to explore the major stressors of farming, coping strategies used by farmers, and their barriers to help-seeking, with the goal of informing suicide prevention and mental health interventions. **Methods:** Fourteen semi-structured interviews were conducted with Kentucky farmers recently receiving a suicide prevention intervention. Participants were purposively sampled and interviewed via telephone or Zoom. Interviews were transcribed, modified verbatim, and then analyzed using reflexive thematic analysis. **Results:** Farmers described chronic stressors, including financial volatility, time scarcity, uncontrollable weather, loneliness, and feeling misunderstood or undervalued by the general public. Despite these stressors, many participants initially denied being stressed. Their coping strategies centered on meaning-making rather than stress elimination, including faith, gratitude, nature-based reflection, occupational pride, and community ties. However, prominent barriers to help-seeking included stigma, self-reliance, and privacy concerns. **Conclusions:** The findings suggest that farmer resilience is primarily enacted through their resourcefulness, including enduring stressors and reframing their thoughts, which enabled their continued functioning despite chronic stress. However, their resourcefulness often obscures their distress and delays help-seeking. These findings may help explain why elevated suicide risk persists among farmers even with strong coping narratives. Interventions that leverage existing strengths while addressing stigma, trust, and structural stressors are needed to better support farmer mental health and suicide prevention.

## 1. Introduction

In 2021, the U.S. age-adjusted suicide rate was 13.44 per 100,000 people [1]. One group with elevated rates of suicide is individuals working in agriculture (i.e., farmers/ranchers). The rate of suicide among non-metropolitan individuals in the U.S. increased from 13.33 per 100,000 in 2001 to 20.20 per 100,000 in 2021 [1], and the rural suicide incidence in Kentucky is higher than the national average, at 19.3 per 100,000 from 2018 to 2024 [2]. Nationally, rural farmworkers have a 32% higher risk of suicide than all other rural occupations [3].

Farmers have several common stressors that affect their mental health and well-being, including financial instability, long working hours, weather uncertainty, geographic and social isolation, and limited access or willingness to use mental health services [4,5,6]. The typical demographic profile of farmer suicide decedents (i.e., older, White, and male) [7,8] aligns with general characteristics of suicide decedents [9]. However, farmer suicide decedents are less likely than the general population of decedents to have had a diagnosed mental health problem or depression before death [8], and in national surveys, farmers have a lower prevalence of reporting poor mental health or depression [10]. These observed patterns, along with pronounced occupational pride and religiosity coupled with persistently elevated suicide mortality [3,11,12], suggest more complex processes between stress exposure, coping, social connectedness, and help-seeking behaviors among farmers.

The interpersonal theory of suicide behavior posits that suicide risk is increased when the factors of perceived burdensomeness, thwarted belongingness, and the capability for suicide converge, alongside an increase in suicidal desire and access to lethal means [13]. Agricultural occupations may amplify these constructs in several ways. Economic pressures and unstable farm incomes may increase feelings of burdensomeness as farmers feel they cannot adequately support their families or sustain an intergenerational farm. Further, geographic and occupational isolation may limit a sense of belonging [14], and occupational access to lethal means and physical risk or habituation to risk may increase the capability for suicide [15]. Meanwhile, self-reliance-focused narratives within agricultural culture may inadvertently increase these dynamics. Resilience framing may intensify farmers’ feelings of burdensomeness and low belongingness by shifting responsibility for coping solely to individuals, rather than addressing the structural causes of their stressors [16]. In farming communities, resilience and stoicism are often valued [17,18], so farmers may interpret their struggles as personal failures rather than as consequences of structural factors, such as those involving markets, policies, and infrastructure. This framing may exacerbate farmers’ feelings of burdensomeness and discourage their help-seeking by reinforcing norms of self-reliance and stoicism [18]. Given these contextual and structural factors, it is critical to understand how farmers currently interpret and manage occupational stress to reduce their suicide risk with appropriate interventions.

The purpose of this paper is to understand the stressors, barriers to seeking help, and the coping strategies used among farmers in the South-Central United States, an area with scant published studies, and to build upon previous research [12] with one-on-one questions seeking to understand how farmers experience coping amid multiple chronic stressors, utilizing a theoretical framework. Framed by the interpersonal theory of suicide, this qualitative study seeks to understand the stressors and barriers to mental health help-seeking and stress coping strategies of farmers. It will provide insight into how farmers experience stress and sustain resilience within the unique cultural, occupational, and environmental context of agriculture by conducting interviews with Kentucky farmers recently receiving a suicide-prevention intervention [19]. With farmers’ perspectives, this analysis aims to inform culturally tailored interventions to mitigate the risk of farmer suicide.

## 2. Methods

The Consolidated Criteria for Reporting Qualitative Research (COREQ) Checklist was used to guide this study [20]. Additional details on methods are available elsewhere, including information on evaluating the challenge coin as a suicide prevention intervention in the farming community [19], which informed the purposive sampling of participants. Participants meeting eligibility requirements (over 18 years of age, English-speaking, received a challenge coin in the last six months, part-time or full-time farmer) were invited to participate in a private Zoom or telephone audio-recorded interview of approximately 30 min. Disclosure of suicidal ideation was not a prerequisite for receiving the suicide prevention intervention [19]; none of the participants expressed mental distress. The interviewer had no prior relationship with any of the participants, but was referred to them by farming community members, who connected them to the research team via email or text. A semi-structured interview guide was utilized to ask the participants questions about their stressors, barriers to seeking help, and methods of coping, as well as their impressions of the suicide prevention intervention. Questions aligned with the interpersonal theory of suicidal behavior, with prompts about feelings of burdensomeness as a farmer and belonging and connection to community. Open-ended questions were also asked to gather more details about contextual factors, community concerns and organizations, and how farmers and their peers are addressing structural factors related to the stressors and burdens that they named (Supplementary Table S1).

The interviews were conducted by the first author, a female PhD researcher with a nursing and rural health background and experience conducting qualitative interviews. Information, including age, race, and marital status, was collected as demographic data. This study was conducted in accordance with the Declaration of Helsinki, and the Institutional Review Board at the University of Southern California approved this study with waived written informed consent due to the minimal risk presented by the study. All participants provided written informed consent for the research, and verbal consent was obtained prior to each interview. After the interviews, each participant received a $25 electronic gift card. The recordings were uploaded to an encrypted storage site and transcribed. Additionally, the interviewer’s field notes were written and uploaded to the study database to enable early idea capture and critical reflexivity. Transcripts and demographic data were then uploaded to Dedoose software (version 9.2.22) [21].

Three researchers (non-farmers with data management, nursing, psychology, and public health backgrounds) read the transcripts and field notes in full to establish codes, or groups of words emerging from the interviews, related to the themes of farmer stressors, barriers to help-seeking, and coping strategies. Interrater reliability of more than 0.80 was established across three interviews between the coders, with discussions about any differences in the codebook [22]. Then the coders separately coded the interviews. The codes were refined as more data emerged from the interviews. Thematic analysis was utilized to analyze the codes emerging from the transcript and the debrief data, and collaborative discussions maintained the reflexivity of the team [23].

Grammarly [24] was utilized to help with writing clarity, but the authors reviewed and verified all changes and take full responsibility for the final manuscript content, data, and conclusions.

## 3. Results

Fourteen interviews with farmers were conducted from March 2024 until June 2024, at which point a reflexive discussion by the research team determined that the dataset provided sufficient depth to address the study aims. The sample age range was 28 to 68 years (*M* = 49.29, *SD* = 12.86), and the mean interview length was 31.4 min. The majority of the sample (86%) was married and identified as male (93%), and all the participants identified as Caucasian non-Hispanic. All participants held farming-related roles, although 77% of the sample also held off-farm roles such as manufacturing. The types of farm production represented in this sample included row crops (e.g., soybeans, corn, and wheat) only (36%), livestock (e.g., beef cattle and sheep) only (29%), livestock and produce (14%), livestock and row crops (14%), and other (7%). Common stressors in farming, barriers to seeking help, and coping strategies used to address stress were described.

The following are themes identified from the transcripts, and illustrative quotes are in Table 1. While none of the participants disclosed suicidal ideation, multiple themes related to the interpersonal theory of suicide, including financial pressure affecting burdensomeness and loneliness, public perception, and limited trust lowering a sense of belonging. Multiple coping factors, such as community activity and faith, were potential protective factors.

### 3.1. Stressors of Farming

The participants shared opinions about stressors that make farming challenging. Several farmers initially indicated they were not stressed, but they proceeded to name numerous stressors. The stressors were driven by finances, a lack of time, uncontrollable weather, loneliness, and feeling misunderstood as farmers.

### 3.2. Financial

Financial concerns, such as making farm payments, were impactful factors creating conditions relevant to burdensomeness, as farmers described thinning margins due to pricing, rising equipment and market prices, and banking concerns. A declining safety net for financial concerns was also mentioned: “Like we had pretty much a guarantee years ago, but we don’t anymore.” (38-year-old). Another farmer (51-year-old) stated: “Everything’s so expensive to buy and rent… ground, equipment’s crazy.” One farmer (64-year-old) described their occupational situation as precarious: “Farmers see it all every day, and they’re striving to make ends meet. And it’s a very thin line between making profit and being able to continue your farming operation and not being able to make a profit and continue your farming operations and have to do something a little different.” Finances were a prominent concern among the sample, particularly among full-time farmers. Farmers who held other employment generally relied on that employment to keep their farms running. One full-time farmer openly wondered: “Am I gonna be able to feed my kids? Make my farm payment?” (64-year-old).

### 3.3. Time Management

Most farmers, especially those with off-farm roles, indicated that time management was difficult, as they missed family events and their partners were stressed by their “second shifts.” As one farmer described his work endurance: “Farming isn’t a Monday-to-Friday job.” (35-year-old). The stress of farming was exacerbated by the sheer number of hours it requires, and they frequently noted that farming is not a job with regular hours or vacations. Multiple participants cited this as preventing farmers from caring for their physical health. Several farmers noted that they cannot “call in sick,” both as a stressor and as a point of pride, characterizing farming as an “essential” occupation. “It’s like the hours that we have to put in sometimes… if I’m sick, I still have to go to work. So that stresses you out. You try to stay healthy so you can be able to do your job every day,” said one 35-year-old farmer, explaining his need to be continually available to work. Other farmers discussed how they could not effectively care for themselves due to the long hours, especially during harvest season, with one (45-year-old) saying “I’m gonna put in long hours and probably not eat right and not take care of myself and not sleep.” This normalized expectation of long hours and little sleep during busy periods was juxtaposed with the need to maintain their health, given the physical and time demands of farming, and it could prevent farmers from being able to lower their burden or engage in coping behaviors, such as connecting with others.

### 3.4. Weather

Weather was noted as a frequent source of stress for farmers, being both unpredictable and entirely beyond their control. Participants frequently described how weather issues interacted with other challenges, such as equipment reliability. One 28-year-old farmer described the following frustrating situation: “You’ll have a breakdown when everything is good weather, weather’s good, ground conditions are good, planning conditions are good, and you may break down. And if it’s a major breakdown or even the slightest, the smallest breakdown, if you can’t get parts in a timely fashion, you may be sitting there, and the rain may be coming in the next day.” Favorable planting, growing, and harvesting periods are brief, which heighten farmers’ urgency to complete tasks and often intensify their stress, also increasing their financial stress, as elaborated by a 35-year-old farmer: “When you look at your workload and think, ‘I’ve gotta get this done,’ and then the weather doesn’t cooperate and all you see is dollar signs going out the window.” Farmers expressed frustration when they could not work due to equipment issues and delays, even when they had an optimal yield. Even minor equipment issues can substantially impede progress, leaving the farmers especially vulnerable to incoming weather. The blend of unpredictable weather patterns and logistical setbacks demonstrated the precarity of farm work and the non-stop farming pressures. One farmer (45-year-old) explained the delicate timing of farming and lack of control facing farmers: “If we screw something up right now, we’re out for 12 months until it’s time to do it again. The thing about farming is we can do everything. We can do it so perfectly, our job’s so perfect that there was no flaw in it whatsoever. And at the end of the day, if Mother Nature didn’t agree with you, or the markets crashed, or something like that, things that are out of our control.”

### 3.5. Loneliness

Many farmers mentioned loneliness, which was associated with the shrinking family farm and the geographic isolation and long hours of farming. Farmers who once worked their farms alongside multiple generations of the family described how farming was now more of a solo endeavor, as family members passed away or chose non-farming occupations. One 51-year-old farmer lamented: “I think that’s one thing, that farmers have a tendency to be alone, and then we grow up working with our dads and our grandpas. And when as they get older and they pass away, then suddenly you don’t have those, your partners in the farm.” They pointed out the extra effort required to connect with others, given the farmers’ far-flung geographic locations and the limited time available after work, a finding that reflected potential thwarted belongingness among farmers. As another farmer (51-years-old) stated about limited time to have relationships, “one of the big dangers of being a farmer is that if you don’t actively develop those relationships and maintain ’em, then you can be alone very quickly.”

### 3.6. They Feel Misunderstood and Undervalued as Farmers

Many farmers in this sample felt that stereotypes tarnished their image and blamed them for harming the environment, even though they aimed to provide food for society, and potentially affecting farmers’ perceived burdensomeness and belongingness. The farmers described the rest of society as undervaluing the occupation. As one farmer (64-year-old) bluntly stated: “Here in the United States, it’s like, oh, that’s just a farmer…. But most people just think, oh, well, he’s just a farmer, and they don’t see the value in what a farmer does,” One farmer (35-year-old) described feeling villainized by environmental groups for farming practices, stating that he believed that “obviously, there’s the hit groups that think we’re worst people in the world killing the earth,” while others described feeling unappreciated and taken for granted, despite long hours and major investments in the farm. Several farmers believed that the general public did not realize farmers’ sacrifices or their low-to-nonexistent profit margins. One farmer (64-year-old) elaborated: “Sometimes, most of the times, farmers really go unappreciated…. if they don’t understand what long hours and hard work farming entails along with all the capital investments and whatnot in farming in order to make a farming venture successful. And most people, when they go to the grocery store, they take the groceries for granted…. They don’t understand that the farmer makes hardly anything.” Respondents generally commented on their perceptions that the general population did not respect their occupation and several felt that they were not cared about, as one farmer (35-year-old) said: “We are a group of people that really feel underappreciated a lot of times.… a lot of farmers think that nobody cares about us.”

### 3.7. Barriers to Reaching Out for Help

The farmer participants shared the barriers keeping them from seeking help in hard times, describing a work ethic that superseded their needs and the “no excuses” mentality of farmers. When time-sensitive tasks were required on the farm, such as harvesting grain or caring for animals, they had no choice but to proceed with the work, ignoring other issues. The tasks on the farm always took priority over their personal issues, even at the peril of their own health. One farmer (45-year-old) explained the urgency of the time window for farmers: “We got a job to do. And that’s what farmers do. We’ve got a time window get out there and do this job at hand when it comes to raising crops, whether it’s harvest, planting season, whatever. So we have to go when it’s time to go.”

Many farmers also described feeling ashamed to bring forward their problems, not wanting to share their burden with others. One participant (47-year-old) described farmers’ independent natures as contributing to stigma and the desire to rely only on themselves, describing “the stigma about being strong, and we do it all ourselves.” (47-year-old). Privacy was another barrier to reaching out for mental health help, as farmers often lived in small communities and did not want their entire towns to know about their mental health. One farmer described himself as “too proud and embarrassed and ashamed a little bit” (68-year-old). “But that’s the problem with farmers. Most of ’em are, are private independent individuals and makes all their decisions for everything they do. And I think, to a high degree, the reason we have such a mental health issue is that they won’t share. They won’t call, reach out. They won’t talk to anybody,” the farmer continued, mentioning the reluctance of farmers to share mental health issues. However, multiple farmers mentioned wanting to be comfortable with whom they disclose information. Most felt like they would selectively trust someone close to them, rather than an unknown professional. According to one 60-year-old farmer: “Most of the farmers I know would fit well into that category as far as you have the pride element and everything else. And not wanting other people around them to know their business and stuff.” Another (40-year-old) stated: “I know a lot of farmers don’t really feel comfortable with just talking to a random person that they don’t know.” One (38-year-old) was not open to seeing a professional they didn’t know: “Me personally, I want to talk to somebody that I’m close to. I probably don’t wanna open up to some counselor…. I’m probably not gonna go to some licensed counselor or something like that…. I would worry about having to reach out to somebody I didn’t know.” These findings supported the view that farmers’ limited disclosure could prevent them from participating in activities that could increase their community connections.

### 3.8. Coping Strategies Among Farmers: Reframing Situations, Reflection and Gratitude, Community and Pride

The participants shared their methods of enduring farm stressors and reducing their perceptions of burdensomeness. Farmers frequently mentioned changing their perspectives by drawing on their grounding in faith, altering their physical environments, and using gratitude strategies. Those who reported that their faiths were helpful to coping with farming stressors held beliefs that life and suffering on Earth are temporary. As one 35-year-old farmer stated, “I think my faith keeps most of everything in perspective. So, I do know that everything here is temporary, and that keeps me focused on what really matters.” Other farmers did not explicitly mention religious faith, but they described reframing difficult situations as temporary, meaningful, or even formative and teaching moments: “What looks so bad turned out to be an absolute fluke blessing,” said one 68-year-old. Many farmers described strategies to intentionally refocus and reframe their thoughts. Another farmer (63-year-old) described how he’s endured hardship over time: “Well, I just take it day at a time, and I, and like I say, I keep coming back to my age and how long I’ve been in the farming. If …. we have a bad day, I keep remembering what my granddad and my dad say, would always say, there’ll be better days. And there always are. It doesn’t take just one bad day to kind of mess everything up or work on you mentally. But you have two or three good days. You come in and you go to bed at night and you get a good night’s sleep. It’s just we just hope that the good days outweigh the bad days.” (63-year-old). Some physically changed their environments, while others reframed their perspectives and even drove around to decompress. A 35-year-old farmer described this: “I’ll get in the truck and just drive around and look at stuff and that seems to help me more than anything.” And another younger farmer (28-year-old) took time away to recharge and change their outlook: “A little downtime in the field, and you get back, get everything fixed, and back going. Once you get it fixed, you realize it could have been worse. I think things could always be worse?”

Several farmers described reflection, gratitude, and moments of solitude as strategies to restore positive thoughts. They might watch their livestock or observe moments in nature such as sunsets, enjoying the quiet time. One 53-year-old farmer enjoyed being present with his cattle: “Sometimes if I can just go home and just be on the farm, just go somewhere on the farm.… just sit down with one of the cows and just be there.” Another farmer reflected upon his life experiences: “Maybe that’s what’s made me who I am today, but it’s obvious that those life moments mold us…. But at the same time, that reflection you have when you stop, and you have something like that’s happening in your life.” (45-year-old). The farmer continued with more about his focus on the positive aspects of his life, even when farming was stressful: “You appreciate a sunset or the sun coming up in the morning or something like that. You think back to stress that you’ve had in your life…. It’s just to be by yourself and have that moment of just don’t wanna say moment of silence, but just to sit back and breathe and appreciate what all has gone right in your life.” Gratitude and hope were frequently mentioned, with farm production frequently viewed with satisfaction: “I look on the bright side of it. I look at all the things that have happened as a result of total as I thought the world was ending kind of thing. And on the other side it’s actually better… the growing process, the producing processes are so satisfying…. every time you had a litter of pigs, it was like a Christmas present. And you just look forward to it. And it’s just one… every day is filled with, with new hope “ (68-year-old). The farmers acknowledged that inherently sad farm events, such as sick animals, were often counterbalanced with joyful ones, such as births.

Most of the farmers mentioned involvement in civic farming community groups, such as cattleman’s associations and Future Farmers of America (FFA). They emphasized the importance of this, describing a close-knit world that they valued, and multiple farmers mentioned how they relied on community groups in addition to their spouses. “We’re just proud of what we do have…. There’s some camaraderie there,” shared another farmer, describing farmers meeting up at different civic groups and also informally (68-year-old). Another farmer (53-year-old) was upbeat about their town: I’ve got a good community of people around me, so that really helps out. The farmers interviewed were proud of their communities and their roles as farmers, helping and feeding their communities and beyond. One (63-year-old) shared a few examples about helping other people in the community with resources from the farm: “Well, it’s just the fact that anything that happens in the community, if there’s a problem, just a minor problem, they will call me…. knowing that you’re doing a good job and helping the community, that really makes me feel good.” Via these groups and individually, occupational pride shone through with many farmers. Not only were they proud of their ambitions, but also of their occupation as an essential, noble role in feeding the nation. “I don’t wanna just make calves. I wanna make good calves,” said one 68-year-old farmer. Another (64-year-old) shared a reminder: “That’s the farmer who’s feeding you and your children.” Many drew pride from being stewards of the land, with feelings of pride and community involvement increasing a sense of belonging. “Crops are living, breathing organisms just like we are. And they require nutrients, they require oxygen, require water all the same things that we require to have a healthy lifestyle. There is a lot of pride in it,” noted one farmer (45-year-old).

## 4. Discussion

Viewed through the interpersonal theory of suicide, this study organized findings relevant to the factors of burdensomeness and belonging by investigating the stressors, barriers to help-seeking, and coping strategies described by one-on-one interviews with farmers only in Kentucky, who work in an occupation with a higher-than-average prevalence of suicide. The present findings build on previous findings about Kentucky and Tennessee farmers describing stressors and a love of farming in focus groups [12]. These data show farmers’ reframing of their hardships as temporary, purposeful, or inherent to farming, which demonstrates individually focused emotional regulation tactics that could conceal their distress, making burdensomeness harder to detect. This may partially explain evidence of farmers having a lower rate of reported mental health distress and diagnosed depression compared to the general population [10], despite a higher rate of suicide [3,8]. These findings add to prior findings suggesting that farmer distress is underrecognized and requires a more nuanced view beyond traditional mental health indicators [10,25]. Similarly, farmers disclose a higher level of religiosity and a lower likelihood of finding suicide as an acceptable action in the face of life’s difficulties than the general population [11], indicating that belief systems may not always translate into timely help-seeking. This study’s sample described typical farming stressors and individual coping techniques, but the participants rarely identified structural or community-level solutions beyond their concerns about public perceptions about farming. This emphasis reinforces cultural norms in agriculture that may prioritize individual responsibility and self-reliance in managing hardships. The present findings illustrate how farmers’ stress may be obscured as they reinterpret hardship as inherent to agricultural work, framing their stress in a culturally reinforced manner.

Echoing prior research [5,26,27], the sample participants identified well-documented stressors. These included time scarcity due to off-farm employment, weather risks, financial uncertainty, and worries about farm sustainability, which increase the conditions where a sense of burdensomeness could increase. Financial stressors, especially commodity and equipment pricing, were frequently mentioned [5]. However, farmer suicide decedent data indicate that financial distress is less commonly documented than that in the general population [8,28]. This suggests that suicide risk may occur via the cumulative interaction of occupational, social, and cultural stressors rather than from financial triggers alone.

One notable finding was that many of the participants initially denied being stressed, despite subsequently describing multiple stressors. This suggests that farmers often continue to function when stressed, concealing issues such as anxiety or depression, which is supported by other literature [29]. This endurance-based resourcefulness may allow farmers to meet the relentless demands of agricultural work, yet it may also obscure psychological distress and delay help-seeking. A clinical implication of this study is that, when working with farmers, providers may reframe questions about stressors and mental health issues as neutral questions about farmers’ occupational situation (i.e., taking a more social determinants approach rather than a direct individual-level mental health symptomology approach).

Participants also reported feeling misperceived and underappreciated by the public [6,15,30]. This finding suggests that greater public outreach on the value of farming and the clarification of misconceptions about farming practices are needed. In the present study, several farmers raised concerns about community gossip, echoing other studies that have found situations in which farmers feel social surveillance [15,30], making trust selective in social support. These findings suggest that a sense of belongingness was uneven among farmers, dependent upon select relationships.

The help-seeking barriers named by the farmers in this study were consistent with those named in other studies, including community gossip, stigma, and self-reliance [4,6,15,26,29,31,32]. The use of mental health services needs to be destigmatized while ensuring privacy, suggesting that telehealth could be promoted to improve access in rural areas. However, despite farmers’ concerns about airing their personal struggles within small towns, many also said they would not want to share issues with an unknown mental health provider. Even local providers could leverage telehealth to further increase confidentiality and trust among farmers.

Stigma in rural areas is a particularly salient factor for epidemiology and suicide prevention. For instance, coroner systems, which are most prevalent in rural areas, show greater misclassification of suicide deaths as undetermined or as accidents [33], potentially out of concerns for the family [34,35]. Other reports have mentioned concerns that farmer suicides may be underreported due to stigma or misclassified as accidental deaths [36,37], potentially due to pressures from insurance exclusionary clauses [38]. However, there is no empirical evidence to support this type of misclassification specifically for farmers, and there are careful protocols for medicolegal death investigations for farming accidents to determine if suicidal intent may have been involved [39].

Many of the farmers in this study reported reframing their perspectives to cope with difficulties by increasing their gratitude for positive aspects of their lives. Gratitude may reduce perceived stress [40] and has been considered an approach to increasing well-being and self-efficacy [41]. Among farmers, some studies have identified mindfulness as a coping strategy or as a means of reframing their situations more positively [4,42]. These findings suggest that farmer-led self-compassion interventions (e.g., self-kindness, gratitude) should be explored [43], although toxic positivity should be avoided, as it can reinforce rural norms of stoicism and discourage farmers from disclosing their feelings [44].

Participants in this study described social support and community ties, all of which involved relying on their partners and peer farmers to discuss stressors, consistent with other studies [4,42,45]. Other studies have shown that most farmers have ample opportunities for social interaction [5,31]. Pride in farming was another theme of this study, consistent with other studies on occupational pride in agriculture, reinforcing purpose [31,32]. Notably, other studies have identified substance use and other numbing activities as coping strategies for farmers [6,42,45], but this was not a finding in this study, suggesting that farming contexts may vary.

Consistent with critiques of resilience perspectives [16], this study’s participants described culturally embedded resilience strategies (faith, pride, community involvement) that counter the overemphasis on individual capacity for adaptation and minimize structural stressors such as market volatility. Pride served a dual role by limiting farmers’ help-seeking yet also uniting them through occupational pride. However, many of the most salient stressors they named (e.g., financial risk, declining safety nets, and weather-related uncertainty, etc.) are largely structural and not readily addressed through individual coping alone. This suggests that interventions framed solely around individual resilience risk overemphasize personal responsibility while underplaying the need for systemic solutions to reduce perceived burdensomeness.

This study was limited by sample size, regional focus, and demographic homogeneity. Additionally, the study sample was predominantly male, which may have affected the findings, especially regarding stoicism, which is often a rural masculine norm [44]. This study was also subject to social desirability bias, as positive behaviors may have been overreported, and negative coping behaviors underreported. Additionally, it is possible that the suicide prevention intervention that participants had completed affected their responses with positive reframing. Several stressors reported in other farmer studies were not reported by members of this study’s sample. For example, other studies identify taxes or healthcare as specific sources of financial stress [5,46,47].

## 5. Conclusions

This study provides additional insight into how farmers experience stress, face barriers to seeking help, and sustain resilience, using the interpersonal theory of suicide as a framework. Farmers described a resourceful process for addressing their chronic stressors, rooted in enduring hardship, pride in farming, faith, reflection, and connection to the land and community. However, agricultural norms that emphasize traits such as self-reliance and pride could contribute to delayed help-seeking and the masking of distress. Future interventions should leverage farmers’ existing strengths, such as gratitude, and trusted peer relationships, while addressing the stressors beyond their individual control. Farmer-initiated, community-centric, and privacy-minded tactics such as telehealth and peer support are potential strategies to enhance agricultural well-being without undermining farmers’ cultural identity.

## Figures and Tables

**Table 1:** Stressors, barriers to help-seeking, and coping strategies employed by farmers, with supporting quotes.

Stressors	Quotes
***Financial***	
Thinning margins, increasing prices	“Everything’s so expensive to buy and rent… ground, equipment’s crazy.” (51-year-old)
	“Farmers see it all every day, and they’re striving to make ends meet. And it’s a very thin line between making profit and being able to continue your farming operation and not being able to make a profit and continue your farming operations and have to do something a little different.” (64-year-old)
	“Am I gonna be able to feed my kids, make my farm payment?” (64-year-old)
Declining safety net	“Like we had pretty much a guarantee years ago, but we don’t anymore.” (38-year-old)
***Time management***	
Need to work around the clock	“Farming isn’t a Monday-to-Friday job.” (35-year-old)
Difficulty maintaining own health	“I’m gonna put in long hours and probably not eat right and not take care of myself and not sleep.” (45-year-old)
	“It’s like the hours that we have to put in sometimes… if I’m sick, I still have to go to work. So that stresses you out. You try to stay healthy so you can be able to do your job every day.” (35-year-old)
***Weather and uncontrollable events***	
Weather and breakdowns	“You’ll have a breakdown when everything is good weather, weather’s good, ground conditions are good, planning conditions are good, and you may break down. And if it’s a major breakdown or even the slightest, the smallest breakdown, if you can’t get parts in a timely fashion, you may be sitting there, and the rain may be coming in the next day.” (28-year-old)
	“When you look at your workload and think, ‘I’ve gotta get this done,’ and then the weather doesn’t cooperate and all you see is dollar signs going out the window.” (35-year-old)
	“If we screw something up right now, we’re out for 12 months until it’s time to do it again. The thing about farming is we can do everything. We can do it so perfectly, our job’s so perfect that there was no flaw in it whatsoever. And at the end of the day, if Mother Nature didn’t agree with you, or the markets crashed, or something like that, things that are out of our control.” (45-year old)
***Loneliness***	
Shrinking family farm	“I think that’s one thing, that farmers have a tendency to be alone, and then we grow up working with our dads and our grandpas. And when as they get older and they pass away, then suddenly you don’t have those, your partners in the farm.” (51-year-old)
Limited time to develop relationships	“One of the big dangers of being a farmer is that if you don’t actively develop those relationships and maintain ’em, then you can be alone very quickly.” (51-year-old)
***Perceived lack of value as farmers***	
Feel undervalued, misunderstood, taken for granted	“Here in the United States, it’s like, oh, that’s just a farmer…. But most people just think, oh, well, he’s just a farmer, and they don’t see the value in what a farmer does,” said one participant (64-year-old).
	“Obviously, there’s the hit groups that think we’re worst people in the world killing the earth,” said another participant, suggesting that farmers feel villainized by environmental groups (35-year-old)
	“Sometimes, most of the times, farmers really go unappreciated.… if they don’t understand what long hours and hard work farming entails along with all the capital investments and whatnot in farming in order to make a farming venture successful. And most people, when they go to the grocery store, they take the groceries for granted…. They don’t understand that the farmer makes hardly anything.” (64-year-old)
	“We are a group of people that really feel underappreciated a lot of times.… a lot of farmers think that nobody cares about us.” (35-year-old)
**Barriers**	
***No-excuses mentality***	
Work ethic as priority	“We got a job to do. And that’s what farmers do. We’ve got a time window get out there and do this job at hand when it comes to raising crops, whether it’s harvest, planting season, whatever. So we have to go when it’s time to go.” (45-year-old)
***Pride, privacy, self-reliance, and shame***	
Independent nature	“too proud and embarrassed and ashamed a little bit…. But that’s the problem with farmers. Most of ’em are, are private independent individuals and makes all their decisions for everything they do. And I think, to a high degree, the reason we have such a mental health issue is that they won’t share. They won’t call, reach out. They won’t talk to anybody,” the farmer continued, mentioning the reluctance of farmers to share mental health issues. (68-year-old)
	“the stigma about being strong, and we do it all ourselves.” (47-year-old).
Selective trust and pride	“Most of the farmers I know would fit well into that category as far as you have the pride element and everything else. And not wanting other people around them to know their business and stuff.” (60-year-old)
	“I know a lot of farmers don’t really feel comfortable with just talking to a random person that they don’t know.” (40-year-old)
	“Me personally, I want to talk to somebody that I’m close to. I probably don’t wanna open up to some counselor…. I’m probably not gonna go to some licensed counselor or something like that…. I would worry about having to reach out to somebody I didn’t know.” (38-year-old)
**Coping strategies**	
***Changing perspectives and reframing***	
Faith and the temporary nature of life	“I think my faith keeps most of everything in perspective. So, I do know that everything here is temporary, and that keeps me focused on what really matters.” (35-year-old)
	“What looks so bad turned out to be an absolute fluke blessing.” (68-year-old)
	“Well, I just take it day at a time, and I, and like I say, I keep coming back to my age and how long I’ve been in the farming. If we have a bad day, I keep remembering what my granddad and my dad say, would always say, there’ll be better days. And there always are. It doesn’t take just one bad day to kind of mess everything up or work on you mentally. But you have two or three good days. You come in and you go to bed at night and you get a good night’s sleep. It’s just we just hope that the good days outweigh the bad days.” (63-year-old)
Changing environments	“I’ll get in the truck and just drive around and look at stuff and that seems to help me more than anything.” (35-year-old)
	“A little downtime in the field, and you get back, get everything fixed, and back going. Once you get it fixed, you realize it could have been worse. I think things could always be worse?” (28-year-old)
***Reflection and gratitude***	
Reflection	“Sometimes if I can just go home and just be on the farm, just go somewhere on the farm.… just sit down with one of the cows and just be there.” (53-year-old)
	“I look on the bright side of it. I look at all the things that have happened as a result of total as I thought the world was ending kind of thing. And on the other side it’s actually better… the growing process, the producing processes are so satisfying…. every time you had a litter of pigs, it was like a Christmas present. And you just look forward to it. And it’s just one… every day is filled with, with new hope.” (68-year-old)
	“Maybe that’s what’s made me who I am today, but it’s obvious that those life moments mold us…. But at the same time, that reflection you have when you stop, and you have something like that’s happening in your life.” (45-year-old)
Gratitude	“You appreciate a sunset or the sun coming up in the morning or something like that. You think back to stress that you’ve had in your life…. It’s just to be by yourself and have that moment of just don’t wanna say moment of silence, but just to sit back and breathe and appreciate what all has gone right in your life.” (45-year-old)
***Community involvement and farmer pride***	
Farmer camaraderie	“I’ve got a good community of people around me, so that really helps out” (53-year-old).
Pride as helpers and farmers	“Well, it’s just the fact that anything that happens in the community, if there’s a problem, just a minor problem, they will call me.… knowing that you’re doing a good job and helping the community, that really makes me feel good” (63-year-old).
	“We’re just proud of what we do have,” stated another (68-year-old)
Pride in cultivating and producing	“I don’t wanna just make calves. I wanna make good calves.” (68-year-old)
	“That’s the farmer who’s feeding you and your children.” (64-year-old)
	“Crops are living, breathing organisms just like we are. And they require nutrients, they require oxygen, require water all the same things that we require to have a healthy lifestyle. There is a lot of pride in it.” (45-year-old).

## Data Availability

The audio recordings and full transcripts from the interviews cannot be shared publicly to protect the participants’ anonymity and confidentiality. Due to the sensitive nature of the interview data and ethical restrictions regarding participant privacy, the full transcripts cannot be made publicly available. De-identified extracts are available from the corresponding author upon reasonable request and with appropriate institutional ethics approval.

## Supplementary Materials

The following supporting information can be downloaded at https://www.mdpi.com/article/10.3390/ijerph23080983/s1. Table S1: Semi-structured interview questions related to farmer stressors, help-seeking, and coping strategies.
