# Supplementary material for: Mental Health Stressors and Barriers to Support Among Rural Farmers: A Qualitative Study of Agricultural Communities in the East South-Central United States
Source: Int J Environ Res Public Health. 2026 Jul 29;23(8):983. doi: 10.3390/ijerph23080983 (PMC13512545; doi:10.3390/ijerph23080983)
Supplement: Supplementary file 1 [file ijerph-23-00983-s001.zip › ijerph-4384664-supplementary.pdf]

Supplementary Table S1.
Semi-structured interview questions related to farmer stressors, help-seeking, and coping strategies

• What is your job title/duties?
• What are some stressors in your role? What makes you feel burdened?
• How do you deal with these? How do your peers?
• Where do you think you'd seek help for mental health if you needed it?
• Where do you think you'd seek help for mental health if you needed it?
• What makes you feel more connected to your community?
• Please share what makes you feel appreciated in your role.
• Is there anything else you would like to mention regarding farmer mental health?
